# A Functional Flatbread (Bazlama): High in Beta-Glucan and Plant-Based Protein Content

**DOI:** 10.3390/foods14030482

**Published:** 2025-02-03

**Authors:** Seda Beyaz, Buket Cetiner, Kubra Ozkan, Osman Sagdic, Francesco Sestili, Hamit Koksel

**Affiliations:** 1 Department of Nutrition and Dietetics, Health Sciences Faculty, Istinye University, Istanbul 34010, Türkiye; beyaz.seda@hotmail.com (S.B.); kubraozkan1907@gmail.com (K.O.); 2 Department of Quality and Technology, Field Crops Central Research Institute, Ankara 06170, Türkiye; buket.cetiner@tarimorman.gov.tr; 3 Department of Food Engineering, Faculty of Chemical and Metallurgical Engineering, Davutpasa Campus, Yildiz Technical University, Istanbul 34349, Türkiye; osagdic@yildiz.edu.tr; 4 Department of Agriculture and Forest Sciences (DAFNE), University of Tuscia, 01100 Viterbo, Italy

**Keywords:** hull-less barley, lentils, traditional bread, phenolics, antioxidants, glycemic index, functional foods

## Abstract

This study focused on developing a functional bazlama with a lower glycemic index (GI) that is high in β-glucan and rich in plant-based protein. Functional bazlama samples were produced by supplementing bread wheat flour with high β-glucan content hull-less barley flour and high protein content lentil flour (15%, 30%, and 45%). Additionally, mixed bazlama samples (Mix1, Mix2, Mix3, and Mix4) were produced by supplementing them with both barley and lentil flours. The results showed that 3 g of β-glucan could be provided from the bazlama sample and supplemented with 45% barley flour, which meets the threshold to carry health claims. Supplementing with 30% and 45% lentil flour increased the protein content of the bazlama samples to a level qualifying them as a “high protein”. The control bazlama had a high GI, while samples supplemented with 30% and 45% barley or lentil flour and all mixed bazlama samples had medium GI values, and Mix2 had the lowest GI value among all bazlama samples. Also, as the supplementation levels of barley and lentil flour increased, the phenolic contents and antioxidant capacities of the bazlama samples increased. The results of the present study indicate that barley and lentils can be used as an ingredient in traditional flatbreads to obtain products with better functional and nutritional properties.

## 1. Introduction

Bread is a fundamental cereal-based food extensively consumed in many different types and forms around the world, supplying a substantial part of daily energy needs [1]. Flatbreads are the oldest and well-known types of bread, which are produced globally in regions such as South Europe, the Middle East, North Africa, Scandinavia, Central America, and part of China [2]. These breads, with origins dating back to ancient times, as evidenced by discoveries from Mesopotamia, ancient Egypt, and the Indus civilization, were likely among the first processed foods [3]. Flatbreads are known by various names worldwide, including baladi, barbari, battaw, bazlama, chapati, ciabatta, hillalla, kalachi, lavash, pita, tandoori, taboon, tortilla, yufka, and many others [4]. These can be produced using wheat, barley, and rye flours or flours obtained from grains such as corn, sorghum, and teff [2].

Bazlama is a type of leavened flatbread with a yellowish creamy crumb color, a creamy crust color with light brown spots, and a uniform diameter and thickness. The crust should be smooth with minimal blisters, while the crumb should have a structure rich in small cells, and the mouthfeel should be enjoyable and easy to chew [5]. Traditionally, refined wheat flour is used to produce bazlama; however partially substituting wheat flour with non-wheat cereal flours or legume flours has been shown to significantly improve the nutritional properties of breads [5,6]. Modern trends highlight the use of alternative flours derived from grains such as barley, rye, and oats, as well as legumes like lentils, chickpeas, and soybean. These flours are rich in protein, dietary fiber, B complex vitamins, minerals, and phytochemicals, offering a healthy alternative to white wheat bread to improve the nutritional value of bread formulations [1,2]. Bread can be fortified to address nutrient deficiencies or serve as a vehicle for compounds that have physiological or nutritional effects, promoting health and contributing to sustainable well-being for its consumers [7].

Barley (*Hordeum vulgare* L.) is one of the oldest cultivated grain crops in the world and holds a significant place in the global total grain production [8]. Barley grain ranks fourth in cereal production, with the annual world harvest amounting to approximately 155 million tons from about 46 million hectares in 2023 [9]. Despite its importance, the majority of barley is used as animal feed and in the malting and brewing industry, with only a very small part used as human food [8,10,11]. However, barley is gaining popularity as a “functional and edible grain” due to its high content of bioactive compounds, such as β-glucan (4–11%), as well as phenolic compounds, minerals, vitamins, and balanced proteins [8,11]. Barley flour can partially or entirely replace wheat flour in various baked goods, including whole-grain bread, unleavened bread, and breakfast cereals. Although not yet widely adopted, barley flour is emerging as a key ingredient in the production of bread, pasta, and biscuits. The addition of barley flour to wheat flour in bread production deteriorates the rheological properties of dough and reduces the volume of bread, which could be due to the lack of gluten in barley [11]. The Food and Drug Administration (FDA) has allowed products containing barley to carry the health claim that they reduce the risk of coronary heart disease, recommending a daily intake of 3 g of β-glucan to help prevent certain diseases, including coronary heart disease [8,12]. Furthermore, a diet rich in β-glucans has a positive effect on human health by preventing chronic non-communicable diseases like cancer, diabetes, and obesity [13].

Lentils (*Lens culinaris*), a nutritious legume cultivated in more than 70 countries, had an annual world harvest of approximately 7 million tons from about 5.7 million hectares in 2023 [9,14]. They contain 21–31% protein and 62–69% carbohydrates [15]. Lentils have approximately twice the protein content of most grains and have a protein levels comparable to meat. The amino acid composition of lentils can complement that of wheat by adding lentils to wheat-based flours, as lentils are particularly high in lysine, leucine, glutamic acid, aspartic acid, and arginine, while grains are rich in sulfur-containing amino acids [15,16,17]. This suggests that consuming lentils and grains together is an effective way of supplying essential amino acid profiles that are well-balanced [16]. Lentil protein shows strong potential as a new functional ingredient in baked goods [18]. Numerous studies have stated the health benefits of consuming lentils, including improved satiety, reduced cholesterol levels, and a lower risk of diet-related chronic diseases such as metabolic syndrome, diabetes, cancer, cardiovascular diseases, and osteoporosis [15,17,19,20]. Compared to other legumes, lentils are rich in phenolics (760 mg GAE/100 g) and exhibit high antioxidant activity [14,15,17]. In addition, lentils have a relatively low glycemic index due to their unique chemical composition, including low starch content, a high amylose/amylopectin ratio, and soluble fibers [16].

Flatbreads have different manufacturing parameters and do not need a wheat flour with very high gluten content/quality and a well-developed gluten network [21,22]. They are less sensitive to gluten content and quality compared to loaf-type breads [22]. Therefore, they have good potential to use whole grain cereal flours or mixed grain flours in their formula. Trends towards using hull-less barley and food legumes as nutritious ingredients in cereal products have been increasing. There are few studies examining the effect of supplementing barley on the quality of bazlama [1,5,22]. However, there remains a lack of studies investigating lentil flour supplementation and the combined effects of barley and lentil flours on bazlama characteristics. This study addresses these research gaps by investigating the potential of barley and lentil flours as ingredients in bazlama production. The aim is to analyze the nutritional and quality properties of bazlama samples produced by partially replacing refined wheat flour with barley and lentil flours. Specifically, the study seeks to produce bazlama with enhanced nutritional properties such as increased β-glucan, protein and phenolic content, higher antioxidant activity, and lower in vitro glycemic index value.

## 2. Materials and Methods

### 2.1. Materials

Bread wheat, hull-less barley, and lentils were used as raw materials in the production of bazlama. Hull-less barley (cv. Chifaa) with a high β-glucan content produced in the 2022–2023 growing season in the city of Marchouch, Morocco, was used. Lentils (Advanced Line) included a high-protein lentil produced in Lebanon. Tosunbey is a hard white bread wheat variety with strong gluten properties, which was produced in the 2021–2022 season, grown in Ankara (Ikizce), Türkiye. The bread wheat (cv. Tosunbey) was obtained from the Field Crops Central Research Institute (TARM); the hull-less barley (cv. Chifaa) was obtained from National Institute of Agricultural Research of Morocco (INRAM); and the lentils (Advanced Line) were obtained from International Center for Agricultural Research in the Dry Areas (ICARDA). Salt, dry yeast (Pakmaya), and granulated sugar were purchased from local markets in Türkiye. The solvents and reagents were all obtained from Sigma-Aldrich (St. Louis, MO, USA). The assay kits for β-glucan and glucose were purchased from Megazyme International (Wicklow, Ireland).

### 2.2. Methods

#### 2.2.1. Milling

Wheat and hull-less barley samples were milled to obtain flour using a Buhler MLU 202 pneumatic laboratory-type mill (Uzwil, Sweden) according to AACCI Method No: 26–21 and 26–31 [23]. Lentil samples were ground to obtain whole lentil flour in a Perten 3100 Laboratory Mill (Perten Instruments, Huddinge, Sweden) according to AACCI Method 26–70.01 [23]. Wheat flour, hull-less barley flour, and whole lentil flour were used for bazlama production.

#### 2.2.2. Chemical Analyses

The moisture contents of flour and bazlama samples were determined according to AACCI Method No: 44-15A [23]. The protein (Nx6.25) content (Leco FP828, St. Joseph, MI, USA) of the bazlama samples was determined according to AACCI Method 46–30 [23].

#### 2.2.3. Farinograph Analysis

Farinograph properties were determined using a Brabender Farinograph-AT (Duisburg, Germany) outfitted with a 50 g bowl according to AACCI Method No: 54-21 [23]. The dough development time (min.), stability (min.), water absorption (14% moisture basis), softening degree (BU, 12 min after the development time), and quality number were determined from Farinograph curves.

#### 2.2.4. Bazlama Production

Bazlama samples were produced by separately supplementing hull-less barley and lentil flours to wheat flour at ratios of 15%, 30%, and 45%. Additionally, to obtain the desired β-glucan and protein ratios, 4 types of mixed bazlama samples were produced using different proportions of wheat, barley, and lentil flours (Mix1: 15% Lentil + 45% Barley + 40% Wheat; Mix2: 15% Lentil + 50% Barley + 35% Wheat; Mix3: 10% Lentil + 50% Barley + 40% Wheat; Mix4: 5% Lentil + 50% Barley + 45% Wheat). Bazlama samples were produced according to the Basman and Koksel [5] with some modifications. During each production, two bazlama samples were obtained. Each sample was produced twice, and finally, four bazlama samples were produced from each flour sample. Two hundred grams of flour (according to 14% moisture basis), dry yeast (2%), salt (1.5%), sugar (1%), and water at 30 °C (according to water absorption value obtained from Farinograph) were used. All ingredients were mixed using a dough mixer (National MFG. Co., Lincoln, Nebraska) according to their Farinograph dough development time until the dough developed. The dough was placed in a fermentation cabinet (Simsek Laborteknik, FK-650, Ankara, Türkiye) with a temperature of 30 °C and a relative humidity level of 85% for 1 h of fermentation. After fermentation, the dough was divided into two equal pieces of approximately 140 g each, rounded by hand, covered with stretch film and left to rest at room temperature for 6 min. Then, it was rolled out into a sheet to a thickness of 7 mm using a rolling pin. The rolled-out dough was baked in a preheated electric skillet (Black&Decker, Baltimore, MD, USA) at 200 °C for 7 min. Bazlama samples were flipped after cooking for 3.5 min, and the other side was baked for another 3.5 min to ensure that both sides were baked equally. After baking, the samples were left to cool at room temperature (22–24 °C). They were placed in plastic bags for bazlama quality evaluation and stored at room temperature (22–24 °C).

#### 2.2.5. Bazlama Quality Evaluations

The L*, a*, b* color values (D65, 10°) of the bazlama samples were determined using a color measurement instrument (MiniScan XE PLUS 45/0-L, Hunter Associates Laboratory Inc., Reston, VA, USA) according to ASTM Method No 1164 [24]. Color readings were made on the crust and the interior of the bazlama samples, and the results were expressed as L* (lightness/darkness), a* (redness/greenness), and b* (yellowness/blueness) values. The total color difference (∆E) was calculated using the following formula [18]:

$$\Delta E=\sqrt{{\Delta L}^{*2}+{\Delta a}^{*2}+{\Delta b}^{*2}}$$

where ∆L: L*sample − L*control; ∆a: a*sample − a*control; and ∆b: b*sample − b*control.

The textural properties of the bazlama samples were analyzed by texture profile analysis (TPA) using a texture analyzer (TA.XT Plus Stable Micro Systems, Surrey, UK) according to the method of Marchetti et al. [25]. The samples were roughly divided into four parts, a single piece was placed on the table of texture analyzer, and four measurements were made for each sample. The parameters of hardness, springiness, cohesiveness, gumminess, chewiness, and resilience were obtained from the TPA curves. Texture profile analyses were performed 2 h, 24 h, and 72 h after production to determine the texture and staling properties of bazlama samples.

#### 2.2.6. Sample Preparations

Bazlama samples were dried in an oven (Mikrotest MST-55, Ankara, Türkiye) at 35 °C for 24 h. Then, the dried bazlama samples were ground using a grinder (Fakir coffee grinder, Istanbul, Türkiye). The ground samples were placed in plastic bags and stored in a refrigerator for further analysis (β-glucan content; protein content; glycemic index; phenolic content; and ABTS, DPPH, and FRAP antioxidant activities).

#### 2.2.7. Beta-Glucan Analysis

β-glucan contents of the bazlama samples were determined according to AACCI Method 32-23.01 [23] using the beta-glucan assay kit (Megazyme International, Wicklow, Ireland).

#### 2.2.8. In Vitro Glycemic Index Value Determination

A glucose assay kit (Megazyme International, Wicklow, Ireland) was used to determine the glycemic index (GI) following the protocols established by Goñi et al. [26] and Tekin-Cakmak et al. [27], respectively.

#### 2.2.9. Determination of Phenolic Contents (Free, Bound, and Total) and Antioxidant Capacities (DPPH, ABTS, and FRAP Methods) of Bazlama Samples

Phenolic compounds (free and bound) of the bazlama samples were extracted according to Shamanin et al. [28]. The Folin–Ciocalteu method was used to determine the concentrations of free and bound phenolic compounds [27]. The results were given as mg of gallic acid equivalent (GAE) per 100 g of dry weight (dw). The total phenolic content (TPC) was calculated as the sum of free and bound phenolics. ABTS, DPPH, and FRAP antioxidant activities were determined according to the methods described in Re et al. [29], Singh et al. [30], and Benzie and Strain [31], respectively. The results were reported as mg Trolox equivalent (TE) per 100 g dry weight (dw).

#### 2.2.10. Statistical Analysis

All experiments were conducted minimum in duplicate, and the mean ± standard deviation was calculated using Excel (Microsoft, Redmond, WA, USA). Results were analyzed using the JMP software (Version 13.2.1, SAS Institute Inc., 2013, Cary, NC, USA). One-way analysis of variance (ANOVA) was applied to evaluate significant differences, and when significant differences were detected (*p* < 0.05), the least significant difference (LSD) method was employed to identify the differences between the means.

## 3. Results and Discussion

### 3.1. Farinograph Properties of Doughs

Knowledge on the rheological properties of dough helps to estimate the wheat flour’s and end product’s quality. The Farinograph analysis results of the control dough and doughs supplemented with barley or lentil flour are presented in Table 1. The control dough exhibited superior Farinograph properties compared to the supplemented samples, demonstrating high stability, a low softening degree, and relatively higher water absorption. These characteristics indicate good bread-making quality, as reported by Cetiner et al. [32]. In contrast, supplementation with barley and lentil flours generally resulted in a deterioration of the dough rheological properties.

As the level of barley flour supplementation increased from 15% to 45%, a clear downward trend was observed in the dough development time, water absorption, stability, and Farinograph quality number values, while the softening degree increased. These findings are consistent with Yu et al. [33], who reported that supplementation of wheat flour with hull-less barley flour (10–40%) negatively impacted the gluten network, resulting in reduced dough development time and stability. The decline in Farinograph properties can depend on different parameters related to the β-glucan, starch, and protein content, as well as the protein quality, as described by Liu et al. [34]. Finocchiaro et al. [35] further corroborated this trend, observing that the enrichment of wheat flour with 30% barley flour reduced dough stability and development time. Interestingly, the water absorption increased with higher barley flour supplementation due to the strong water-binding properties of the β-glucans.

The supplementation of lentil flour also negatively affected the dough’s Farinograph properties when compared to the control. Specifically, the lentil flour significantly increased the softening degree and reduced dough stability, which is consistent with the findings of Turfani et al. [36], who attributed these effects to the weakening of the gluten network caused by legume flours. The addition of lentil flour to wheat flour dilutes the gluten content, and it deteriorates the gluten network and rheological properties of dough mainly due to the lack of gluten in lentils. Additionally, our results align with those of Cacak-Pietrzak et al. [37], who observed that the softening degree increased proportionally with the level of lentil flour supplementation. In summary, both barley and lentil flour supplementation deteriorated the rheological properties of the wheat flour dough, particularly with respect to lower gluten content, dough stability, and softening behavior. These changes are influenced by the composition of the supplemented flours, including β-glucans, proteins, and starches, which interact with the gluten network and water absorption capacity.

### 3.2. Textural Profile Analysis of the Bazlama Samples

The hardness, springiness, cohesiveness, gumminess, chewiness, and resilience characteristics of the bazlama samples at the 2nd hour, 24th hour, and 72nd hour of storage are given in Table 2. The hardness, gumminess, and chewiness had a generally negative correlation with the bread quality and a positive correlation with cohesiveness and springiness [38]. Compared to the control sample, there were no statistically significant differences in the hardness values of the bazlama samples with 15% barley flour supplementation throughout the storage period. It can be stated that the bazlama texture was not affected significantly with the 15% barley flour supplementation. Differently, the hardness values of the bazlama samples significantly increased with the supplementation of 30% and 45% barley or lentil flour (*p* < 0.05). Furthermore, the TPA results indicated that the hardness values of barley flour supplemented bazlama samples were much lower than those of lentil flour supplemented bazlama samples. Compared to the control bazlama, the hardness of all mixed bazlama samples (Mix1, Mix2, Mix3, and Mix4) increased, with Mix4 having the lowest hardness value among them. The hardness of the bazlama samples supplemented with barley flour increased significantly during the storage period (*p* < 0.05). The hardness of the bazlama samples with lentil flour supplementation increased significantly during the storage period, except for the bazlama sample with 45% lentil flour supplementation (*p* < 0.05). The hardness of all mixed bazlama samples also increased significantly at the 2nd, 24th, and 72nd hours of storage (*p* < 0.05). To sum up, the hardness values of the bazlama samples generally increased during three days of storage (*p* < 0.05). This was related not only with starch crystallization and retrogradation but also with water loss during storage. In this regard, bread staling presents a challenge for preserving the quality of baked goods [39]. Similarly, several other studies in the literature reported that substituting wheat flour with barley flour or legume flour caused a statistically significant increase in the bread crumb hardness [34,37,40]. When making bread, replacing a substantial portion of wheat flour with non-gluten-forming flours like barley or lentil flour will significantly reduce the dough’s viscoelasticity and the gas retention capacity of the blended dough matrices. Weakened gluten structures often lead to bread with decreased volume, poorer texture, altered appearance and color, and lower sensory quality. Lower gas-holding capacity and volume can be a significant quality problem in loaf-type bread. However, flatbreads such as bazlama are more tolerant to changes in gluten quality and quantity, as they have different quality evaluation criteria compared to loaf-type breads [22]. Therefore, they have good potential to use whole grain cereal flours or mixed grain flours in their formula. Although a formal sensory analysis was not performed (due to requirement of ethics committee permission), all of the bazlama samples produced in the present study (supplemented with barley/lentil flour and the mixed samples) had acceptable sensory properties.

Cohesiveness, springiness, and resilience are three crucial textural properties impacted mainly by gluten quality. Low cohesiveness indicates that the crumb is highly prone to breaking or crumbling, negatively impacting the consumer acceptance of bread [41]. There were no significant differences between the cohesiveness values of the bazlama samples supplemented with barley or lentil flour compared to the control bazlama sample at the 2nd and 24th hours of storage. However, at the 72nd hour, a significant decrease was observed in general compared to the control bazlama sample (*p* < 0.05). Supplementing with barley and lentil flours generally reduced the cohesiveness values of the mixed bazlama samples (*p* < 0.05). The cohesiveness values of all bazlama samples generally decreased during the storage period, except for the bazlama sample with 45% lentil flour supplementation (*p* < 0.05).

The difference between springiness values of bazlama samples supplemented with barley flour was not statistically significant compared to the control bazlama at the 2nd hour, 24th hour, and 72nd hour of storage (*p* < 0.05). Supplementing barley and lentil flours together led to a decrease in the springiness of the mixed bazlama samples (*p* < 0.05). Yu et al. [33] concluded that the springiness values of bread supplemented with hull-less barley did not change significantly. Cacak-Pietrzak et al. [37] stated that addition of lentil flour caused a consistent decrease in the springiness values compared to the control bread. The results obtained by Yu et al. [33] and Cacak-Pietrzak et al. [37] support the findings of the present study. Springiness and resilience are often linked, and a reduction in these parameters is associated with a decline in crumb elasticity [33,41]. Compared to the control bazlama sample, the resilience values of the bazlama samples supplemented with barley or lentil flour did not have significant differences, except for the 45% lentil flour supplemented bazlama sample (at the 2nd and 72nd hours). The resilience values of the mixed bazlama samples generally had a decreasing trend compared to the control bazlama sample at the 2nd and 24th hours of storage, while no statistically significant difference was observed at the 72nd hour (*p* < 0.05).

The chewiness values were generally increased gradually during storage at room temperature for up to 72 h. As the percentage of barley flour supplementation increased, the chewiness also generally increased (*p* < 0.05). A similar trend was observed in the lentil flour supplemented bazlama samples. These results are in line with the findings of Koksel et al. [22], who showed that the chewiness of all bazlama samples increased throughout the 48-h storage period at room temperature. Schmidt et al. [42] concluded that the increase in hardness and chewiness indicated that bread enriched with β-glucan had a denser and more resilient crumb that adhered to the teeth, making it more difficult to chew. The gumminess of all bazlama samples generally increased during storage at room temperature for up to 72 h (*p* < 0.05). In the study conducted by Liu et al. [34], 20% wheat flour and 80% normal hull-less barley (HLB) whole grain flour (WGF) were used in bread making. Adding HLB WGF to wheat breads resulted in a noticeable increase in the hardness, gumminess, and chewiness while reducing the cohesiveness. Finocchiaro et al. [35] confirmed with their rheological findings that the overall quality of bread slightly worsened when barley flour was added to the dough. They stated that the main reason for this situation was the insufficient gluten concentration in the dough. Nkurikiye et al. [40] found that increasing the inclusion level of legume flours did not significantly affect the resilience, cohesiveness, springiness, and gumminess, but it did lead to a significant increase in chewiness.

### 3.3. Crumb and Crust Color Properties of the Bazlama Samples

**Figure 1:**
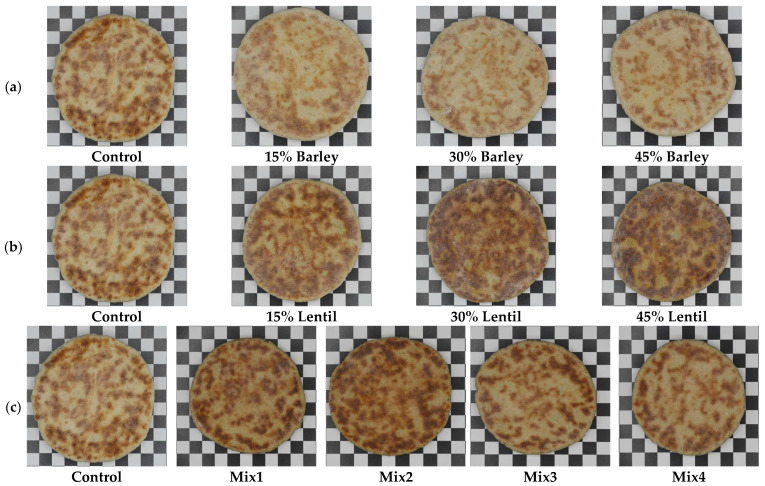
Bazlama samples supplemented with (**a**) barley flour and (**b**) lentil flour; (**c**) Mix1, Mix2, Mix3, and Mix4 bazlama samples produced by using wheat, barley, and lentil flours. (Mix1: 40% Wheat + 45% Barley + 15% Lentil; Mix2: 35% Wheat + 50% Barley + 15% Lentil; Mix3: 40% Wheat + 50% Barley + 10% Lentil; Mix4: 45% Wheat + 50% Barley + 5% Lentil).

The color values of the bazlama samples are given in Table 3. The supplementation with barley (β-glucan) and lentils flours (protein) affected the color of the bazlama samples (Figure 1). The L* values of the bazlama crumb decreased with increasing levels of barley flour incorporation (*p* < 0.05). In agreement with our findings, Kurek et al. [39] reported that the brightest crumb color was found in the control group (70.65), while the L* value decreased (66.07) in the barley flour supplemented bazlama. Similarly, Koksel et al. [22] reported that the crumb and crust L* values decreased as the barley flour supplementation level increased in their bazlama samples. As the lentil flour supplementation level increased, the crumb and crust L* values decreased in the bazlama samples. Among the 11 types of bazlama, the sample with 45% lentil flour supplementation had the lowest L* value for both the crumb and crust. Consistent with the present study, it has been observed that higher proportions of legume flours in flour blends lead to darker crusts in baked products. This crust darkening was attributed to the increased Maillard reaction during baking due to the higher lysine content in legumes. In the Maillard reaction, reducing carbohydrates react with free amino acid side chains of proteins, primarily lysine, producing brown pigments as by-products of the amino acid–sugar reactions [43].

Compared to the control, as both the barley flour and lentil flour supplementation level increased, the crumb a* color values increased, while there were no significant differences in the crust a* values. On the other hand, in a study by Kurek et al. [39], the crust color a* value was significantly higher in the control bread, while this value decreased in the barley-added bread. With the supplementation of barley flour, no significant differences were observed in the crumb b* values; however, the crust b* values generally decreased as the barley flour level increased (*p* < 0.05). Bazlama samples supplemented with lentil flour had higher crumb b* yellowness values than the control, which is a feature generally desired by consumers. However, the crust b* color value decreased with the lentil flour supplementation, but there were no significant differences among the lentil flour supplemented bazlama samples.

The total crumb color difference of barley- and lentil-flour-supplemented bazlama samples ranged from 3.91 to 11.66 and 12.68 to 19.30, respectively. The total crust color difference of barley- and lentil-flour-supplemented bazlama samples ranged from 5.69 to 7.87 and 5.16 to 9.93, respectively. The ∆E values increased with the increasing levels of both barley and lentil flours in the crumb and crust color. Perceivable color differences can be analytically categorized as small (1.5 < ΔE), distinct (1.5 < ΔE < 3), and very distinct differences (ΔE > 3) [44]. According to this classification, it can be seen that the total color differences in all bazlama samples are very distinct. Therefore, the ΔE value indicated an important effect of barley and lentil flours addition on the color of the bazlama.

### 3.4. Protein and β-Glucan Contents; In Vitro GI Values of the Bazlama Samples

Foods can be categorized as low (GI ≤ 55), medium (GI 56–69), or high (GI ≥ 70) glycemic index foods according to their GI values [45]. The in vitro GI values of the bazlama samples are given in Table 4. The in vitro GI values of the bazlama samples supplemented with barley and lentil flour ranged from 62.27 to 71.30 and 61.78 to 70.50, respectively, compared to that of the control bazlama (75.16). Notably, the in vitro GI values of the barley- or lentil-flour-supplemented bazlama samples significantly decreased as the barley or lentil levels increased (*p* < 0.05). Decreased in vitro GI values were also observed in mixed bazlama samples containing both barley and lentil flours. The lowest values were obtained in the Mix2 (57.24) and Mix3 (58.01) bazlama samples. The bazlama samples supplemented with 30% and 45% barley or lentil flour and all the mixed bazlama samples shifted from high-GI foods to medium-GI foods. Kazemi et al. [46] encouraged the consumption of barley bread due to its potential to lower overall GI values in the Iranian diet. Similarly, Thondre and Henry [47] demonstrated that barley β-glucan significantly reduced the GI of chapatis. Several studies concluded that the consumption of barley or lentil may help to reduce the postprandial glycemic response [20,48].

The β-glucan contents of the control bazlama and the bazlama samples supplemented with barley and lentil flours are presented in Table 4. The β-glucan content of the control bazlama sample was determined to be 0.43 g/100 g on a dry basis, while the β-glucan contents of the bazlama samples supplemented with barley flour were between 0.86 and 1.85 g/100 g on a dry basis. The β-glucan contents of the barley-supplemented bazlama samples significantly increased as the level of barley flour in the bazlama formulation increased (*p* < 0.05). Bread consumption has been comparatively higher in Türkiye and several other Mediterranean countries, typically between 200–250 g per day [22]. Based on the bread consumption levels in East and South Mediterranean countries, it is estimated that consuming 200–250 g per day of bazlama sample prepared with 45% barley flour supplementation could provide approximately 2.41 ± 0.02–3.01 ± 0.03 g of β-glucan, respectively. It is estimated that consuming 200–250 g per day of the Mix1, Mix2, Mix3, and Mix4 bazlama samples, containing both barley and lentil flours, could provide approximately 1.89–2.36 g, 1.97–2.46 g, 2.02–2.53 g, and 2.10–2.63 g of β-glucan, respectively. Based on these results, the bazlama sample supplemented with 45% barley flour, with a consumption of 250 g, meet the requirements to carry a health claim by providing the necessary daily amount of β-glucan (3 g). The β-glucan contents of the bazlama samples supplemented with lentil flour decreased significantly, as was expected (*p* < 0.05). As the lentil flour supplementation increased from 15% to 45%, the β-glucan content decreased from 0.37 g/100 g to 0.14 g/100 g on a dry basis.

The protein contents of the control, barley-flour-supplemented, lentil-flour-supplemented, and mixed bazlama samples are provided in the Table 4. There were significant differences between the bazlama samples regarding protein content (*p* < 0.05). Partially replacing wheat flour with lentil flour led to a statistically significant increase in protein content (*p* < 0.05). The protein contents of the 15, 30, and 45% lentil-flour-supplemented bazlama samples were in the range of 18.23–22.15%, while that of the control bazlama was 16.67%. Since all legumes have a higher protein content compared to wheat flour, supplementing legume flour to wheat flour can enhance the nutritional value of foods. The protein content of the bazlama samples generally decreased as the level of barley flour supplementation increased (*p* < 0.05). As the barley flour level increased to 45%, the protein content decreased from 16.67% to 14.59%. Mix1, containing 15% lentil flour, had the highest protein content among all the mixed bazlama samples. Carcea et al. [49] reported that lentil-supplemented bread had a 30% higher protein content compared to wheat bread, as well as a more balanced amino acid profile. Lentil flour supplementation enhances the level of nearly all essential amino acids in bread, since the amino acid profiles of wheat and lentil complement each other [49].

Assuming a daily consumption of 200–250 g of bazlama supplemented with lentil flour, the protein amounts obtained from the samples with 15%, 30%, and 45% lentil flour supplementation would be 23.7–29.6 g, 26.3–32.9 g, and 28.8–36 g, respectively. According to Regulation (EC) No 1924/2006 [50] of the European Parliament and Council on Nutrition and Health Claims Made on Foods, a food can be labeled as a “source of protein” if protein contributes at least 12% of its energy value, and “high protein” if protein accounts for at least 20% of its energy value. The energy amount was calculated according to the Guidelines on Nutrition Labelling [51] by using the following conversion factors: a carbohydrate content of 4 kcal/g, a protein content of 4 kcal/g, and a fat content of 9 kcal/g. Since the fat content of wheat flour and lentil is quite low, the fat content of bazlama samples was assumed to be 1% in the energy calculation [52]. The results showed that protein contributed 18.00%, 20.00%, and 21.85% of the total energy in bazlama samples supplemented with 15%, 30%, and 45% lentil flour, respectively. Notably, in the sample with 30% lentil flour, 20% of the energy comes from protein, while in the sample with 45% lentil flour, the protein contribution exceeds 20%. Therefore, under Regulation (EC) No 1924/2006 [50], the nutritional claim “high protein” can be applied to bazlama samples supplemented with 30% and 45% lentil flour.

### 3.5. Phenolic Contents and Antioxidant Capacities of the Bazlama Samples

Phenolic compounds are effective antioxidants because they scavenge reactive radicals, inhibit lipid peroxidation, and chelate iron [53]. Phenolic compounds exist in free and bound forms in cereals and food legumes [54]. The phenolic contents and antioxidant capacity values (free, bound, and total) of the bazlama samples are presented in Table 5. The total phenolic content (TPC) of the control bazlama sample was determined as 405.06 mg GAE/100 g dw, while the TPCs of the bazlama samples supplemented with barley flour were significantly higher and ranged from 442.66 to 475.29 mg GAE/100 g dw. Most of the phenolics were found in the bound form, as was expected. Compared to the control bazlama sample, the bazlama supplemented with 45% barley flour had the highest amount of bound phenolics (243.95 mg/100 g dw), followed by the bazlama samples supplemented with 30% (235.54 mg/100 g dw) and 15% (232.25 mg/100 g dw) barley flour. As the supplementation level of the barley flour increased, the free and bound phenolic contents increased significantly compared to the control sample (*p* < 0.05). In the study conducted by Sharma and Gujral [55], barley flour was added to wheat flour at the levels of 28%, 56%, and 84%, and significant increases in chapatti TPC values of 26.5%, 49.5%, and 57.1% (*p* ≤ 0.05) were achieved, respectively. del Carmen Robles-Ramírez et al. [56] concluded that the replacement of wheat flour with 60% barley flour increased the breads’ total phenolic content by 41.5% and the antioxidant activity by 45%. The results of Sharma and Gujral [55] and del Carmen Robles-Ramírez et al. [56] are in agreement with the results of the present study.

It has been reported that lentils have a high content of phenolic compounds and show high antioxidant activity [49]. The total phenolic contents of the bazlama samples supplemented with lentil flour were significantly higher than the control bazlama sample, ranging from 419.68 to 463.36 mg GAE/100 g dw. Like in the barley-supplemented bazlama samples, the majority of the phenolics were also in the bound form. As the level of lentil flour supplementation increased in the bazlama samples, the free and bound phenolic compounds increased significantly compared to the control bazlama sample (*p* < 0.05). Similar to the present study, other studies stated that significantly higher levels of phenolic compounds, especially those found in the aqueous organic extract, were obtained in legume-flour-supplemented bread [37,49]. The TPCs of the mixed bazlama samples supplemented with barley and lentil flours were significantly higher than the control bazlama sample and ranged between 491.16 and 507.12 mg GAE/100 g dw. Among all the mixed bazlama samples, Mix2 had the highest TPC value. Compared to the control bazlama sample, the Mix2 (243.95 mg/100 g dry weight) and Mix3 (241.68 mg/100 g dry weight) bazlama samples had the highest amounts of free phenolics, while the Mix1 (260.60 mg/100 g dry weight) and Mix2 (263.17 mg/100 g dry weight) bazlama samples had the highest amounts of bound phenolics. As the barley and lentil flour supplementation levels increased, the free and bound phenolic contents increased significantly compared to the control bazlama sample (*p* < 0.05).

The antioxidant capacities of cereal and cereal products are typically evaluated using multiple methods due to their complex structures [22]. In the present study, three methods (ABTS, DPPH, and FRAP) were used to estimate antioxidant capacity. The DPPH, FRAP, and ABTS values of the free and bound forms are given in Table 5. As the supplementation levels of the barley and lentil flour increased in the bazlama formulation, the antioxidant capacities for both the free and bound forms significantly increased in all three methods (DPPH, FRAP, and ABTS) compared to the control bazlama sample (*p* < 0.05). The total DPPH values of bazlama samples supplemented with barley flour ranged from 87.14 to 115.36 mg TE/100 g dw compared to 73.05 mg TE/100 g dw in the control bazlama. For samples supplemented with lentil flour, the total DPPH values varied between 84.68 and 104.15 mg TE/100 g dw. In the mixed bazlama samples, the total DPPH values were notably higher, ranging between 148.72 and 180.31 mg TE/100 g dw, with Mix2 exhibiting the highest value.

The total FRAP values of the bazlama samples supplemented with barley flour ranged from 66.35 to 103.26 mg TE/100 g dw compared to the total FRAP values of the bazlama samples supplemented with lentil flour that ranged from 65.48 to 101.74 mg TE/100 g dw. The control group had the lowest total FRAP value (49.80 mg TE/100 g dw). In other words, with the increase in both the barley and lentil flour supplementation levels, the FRAP values of bazlama samples increased significantly (*p* < 0.05). Similar results were also reported in the literature. In wheat–lentil bread, approximately a quarter of the wheat flour was replaced with lentil flour, and the antioxidant power measured by FRAP was significantly higher than in wheat bread [49]. The total FRAP values of the mixed bazlama samples ranged from 106.12 to 137.36 mg TE/100 g dw. Mix2 was the bazlama sample with the highest FRAP value for both free (59.86 mg TE/100 g dw) and bound (77.50 mg TE/100 g dw) forms, with a total FRAP value of 137.36 mg TE/100 g dw.

The control group had the lowest total ABTS value (138.14 mg TE/100 g dw), while the total ABTS values of the bazlama samples supplemented with barley flour ranged from 236.25 to 567.50 mg TE/100 g dw, and those supplemented with lentil flour ranged from 232.52 to 437.30 mg TE/100 g dw. The ABTS values of the bazlama samples increased significantly with the respective increases in the supplementation levels of the barley and lentil flours (*p* < 0.05). Holtekjølen et al. [10] observed that the antioxidant properties of bread produced by replacing 40% of wheat flour with barley flour increased compared to the control bread. In a recent study, the incorporation of lentil flour into bread (10%, 15%, 20%, and 25%) demonstrated a more remarkable ability to scavenge ABTS and DPPH radicals, which increased the bread’s bioactive compound content and antioxidant activity [37]. The total ABTS values of the mixed bazlama samples were between 568.66 and 595.50 mg TE/100 g dw. Mix2 and Mix3 had the highest ABTS values for both free and bound forms, with total ABTS values of 595.50 and 593.92 mg TE/100 g dw, respectively. The DPPH, FRAP, and ABTS values of the bazlama samples supplemented with barley and lentil flours demonstrated their potential to enhance the antioxidant capacities of bazlama.

## 4. Conclusions

Consumers are becoming more conscious of the connection between nutrition and diseases, driving a global focus on a balanced diet and the development of healthier, more nutrient-dense, and fortified functional foods. This study redesigned the traditional flatbread bazlama, using high β-glucan barley and high-protein lentils, to enhance its health benefits. Bazlama samples supplemented with 30% or 45% barley or lentil flour and all mixed bazlama samples shifted from high-GI foods to medium-GI foods, and the nutritional claim “high protein” can be applied to the labeling of the bazlama samples with 30% and 45% lentil flour supplementation. Consuming 250 g per day of bazlama sample prepared with 45% barley flour supplementation could provide approximately 3 g of β-glucan, which the FDA recommends as a daily intake of that amount to help prevent certain diseases, including coronary heart disease.

By supplementing barley and lentil flours to bazlama samples, the β-glucan and protein contents increased, the glycemic index values decreased, and the phenolic content and antioxidant capacities were enhanced, resulting in functional food products with improved health benefits. Accordingly, barley and lentils can be used as a good source of β-glucan and protein in bazlama production. Incorporating barley and lentil flours into traditional foods not only meets consumer demand for functional and healthier products but also promotes the use of sustainable and nutrient-rich ingredients. This approach offers a practical pathway to addressing public health concerns related to diet-related chronic diseases. Further research could explore sensory properties and consumer acceptance, ensuring the successful integration of these functional ingredients into food products.

## Figures and Tables

**Table 1:** Farinograph results of doughs supplemented with barley or lentil flour.

Sample	Development Time (min)	Water Absorption (%)	Stability (min)	Softening Degree ^1^ (BU)	Quality Number
Control	6.50 ± 0.18 ^a^	61.6 ± 0.21 ^a^	11.92 ± 0.02 ^a^	45 ± 1 ^c^	148 ± 4 ^a^
15% Barley	4.68 ± 0.28 ^b^	60.5 ± 0.07 ^b^	9.34 ± 0.13 ^b^	59 ± 1 ^bc^	105 ± 0 ^b^
30% Barley	1.39 ± 0.04 ^c^	59.1 ± 0.64 ^c^	7.37 ± 0.69 ^c^	61 ± 7 ^b^	78 ± 7 ^c^
45% Barley	1.25 ± 0.10 ^c^	57.5 ± 0.28 ^d^	2.98 ± 0.42 ^d^	93 ± 8 ^a^	32 ± 5 ^d^
Control	6.50 ± 0.18 ^a^	61.6 ± 0.21 ^a^	11.92 ± 0.02 ^a^	45 ± 0.7 ^c^	148 ± 3.5 ^a^
15% Lentil	4.38 ± 0.15 ^c^	61.8 ± 0.28 ^a^	4.14 ± 0.41 ^b^	107 ± 13.4 ^b^	71 ± 4.9 ^bc^
30% Lentil	4.58 ± 0.05 ^c^	60.1 ± 0.42 ^b^	2.21 ± 0.03 ^c^	151 ± 1.4 ^a^	62 ± 1.4 ^c^
45% Lentil	5.73 ± 0.22 ^b^	57.1 ± 0.14 ^c^	2.21 ± 0.14 ^c^	118 ± 7.8 ^b^	76 ± 2.1 ^b^

The statistical analysis was performed separately within each group, and values followed by different letters in the same column for each group (barley-supplemented and lentil-supplemented bazlama samples) are significantly different (*p* < 0.05). ^1^ 12 min after the development time.

**Table 2:** Textural properties of the bazlama samples.

Sample	Hardness (N)	Springiness	Cohesiveness	Gumminess	Chewiness	Resilience
**2 h**						
Control	1.05 ± 0.04 ^cC^	0.974 ± 0.021 ^aA^	0.81 ± 0.05 ^aA^	0.85 ± 0.05 ^cC^	0.83 ± 0.06 ^cC^	0.401 ± 0.009 ^aA^
15% Barley	1.09 ± 0.09 ^cC^	1.032 ± 0.117 ^aA^	0.81 ± 0.02 ^aA^	0.88 ± 0.08 ^cC^	0.90 ± 0.08 ^cC^	0.398 ± 0.008 ^aA^
30% Barley	1.91 ± 0.12 ^bC^	0.964 ± 0.012 ^aA^	0.78 ± 0.01 ^aA^	1.50 ± 0.08 ^bC^	1.44 ± 0.06 ^bC^	0.393 ± 0.010 ^aA^
45% Barley	2.24 ± 0.20 ^aC^	0.956 ± 0.021 ^aA^	0.77 ± 0.03 ^aA^	1.72 ± 0.10 ^aC^	1.65 ± 0.13 ^aC^	0.395 ± 0.019 ^aA^
Control	1.05 ± 0.04 ^dC^	0.974 ± 0.021 ^aA^	0.81 ± 0.05 ^aA^	0.85 ± 0.05 ^dC^	0.83 ± 0.06 ^cC^	0.401 ± 0.009 ^aA^
15% Lentil	1.98 ± 0.18 ^cC^	0.938 ± 0.014 ^bcB^	0.82 ± 0.01 ^aA^	1.63 ± 0.15 ^cC^	1.53 ± 0.15 ^cC^	0.399 ± 0.002 ^aA^
30% Lentil	3.11 ± 0.44 ^bC^	0.973 ± 0.028 ^abA^	0.79 ± 0.01 ^aA^	2.44 ± 0.34 ^bC^	3.02 ± 0.72 ^bB^	0.386 ± 0.009 ^aA^
45% Lentil	6.50 ± 0.50 ^aA^	0.930 ± 0.021 ^cA^	0.75 ± 0.04 ^aA^	4.62 ± 0.67 ^aB^	4.28 ± 0.54 ^aA^	0.359 ± 0.029 ^bA^
Control	1.05 ± 0.04 ^dC^	0.974 ± 0.021 ^aA^	0.81 ± 0.05 ^aA^	0.85 ± 0.05 ^dC^	0.83 ± 0.06 ^dC^	0.401 ± 0.009 ^abA^
Mix1	4.43 ± 0.61 ^bC^	0.927 ± 0.014 ^cA^	0.75 ± 0.03 ^bA^	3.30 ± 0.42 ^bB^	3.06 ± 0.43 ^bB^	0.366 ± 0.017 ^cA^
Mix2	6.01 ± 0.13 ^aC^	0.930 ± 0.009 ^bcA^	0.73 ± 0.01 ^bA^	4.39 ± 0.12 ^aC^	4.08 ± 0.09 ^aC^	0.357 ± 0.003 ^cA^
Mix3	4.26 ± 0.35 ^bC^	0.944 ± 0.023 ^bcA^	0.76 ± 0.01 ^abA^	3.25 ± 0.23 ^bC^	3.07 ± 0.22 ^bC^	0.386 ± 0.015 ^bA^
Mix4	2.61 ± 0.04 ^cC^	0.954 ± 0.012 ^abA^	0.79 ± 0.01 ^aA^	2.07 ± 0.03 ^cC^	1.98 ± 0.01 ^cC^	0.418 ± 0.011 ^aA^
**24 h**						
Control	1.88 ± 0.34 ^bB^	0.970 ± 0.013 ^aA^	0.76 ± 0.03 ^aA^	1.42 ± 0.23 ^bB^	1.38 ± 0.24 ^bB^	0.368 ± 0.011 ^aB^
15% Barley	2.23 ± 0.11 ^bB^	0.962 ± 0.016 ^aA^	0.74 ± 0.01 ^aB^	1.65 ± 0.10 ^bB^	1.58 ± 0.07 ^bB^	0.358 ± 0.012 ^aB^
30% Barley	3.81 ± 0.10 ^aB^	0.965 ± 0.010 ^aA^	0.72 ± 0.02 ^aB^	2.75 ± 0.02 ^aB^	2.75 ± 0.17 ^aB^	0.364 ± 0.012 ^aB^
45% Barley	3.86 ± 0.73 ^aB^	0.958 ± 0.015 ^aA^	0.73 ± 0.03 ^aA^	2.93 ± 0.46 ^aB^	2.51 ± 0.42 ^aB^	0.385 ± 0.031 ^aA^
Control	1.88 ± 0.34 ^dB^	0.970 ± 0.013 ^aA^	0.76 ± 0.03 ^aA^	1.42 ± 0.23 ^dB^	1.38 ± 0.24 ^dB^	0.368 ± 0.011 ^aB^
15% Lentil	3.45 ± 0.59 ^cB^	0.963 ± 0.007 ^aA^	0.74 ± 0.02 ^aB^	2.54 ± 0.38 ^cB^	2.45 ± 0.38 ^cB^	0.361 ± 0.013 ^aB^
30% Lentil	4.88 ± 0.38 ^bB^	0.968 ± 0.012 ^aA^	0.74 ± 0.03 ^aB^	3.59 ± 0.16 ^bB^	3.47 ± 0.19 ^bAB^	0.374 ± 0.028 ^aA^
45% Lentil	8.69 ± 0.54 ^aA^	0.966 ± 0.057 ^aA^	0.73 ± 0.06 ^aA^	6.77 ± 0.54 ^aA^	6.53 ± 0.39 ^aA^	0.344 ± 0.041 ^aA^
Control	1.88 ± 0.34 ^eB^	0.970 ± 0.013 ^aA^	0.76 ± 0.03 ^aA^	1.42 ± 0.23 ^eB^	1.38 ± 0.24 ^dB^	0.368 ± 0.011 ^aB^
Mix1	8.70 ± 0.48 ^aB^	0.926 ± 0.014 ^bcA^	0.66 ± 0.02 ^bcB^	5.52 ± 0.14 ^aA^	5.06 ± 0.12 ^aA^	0.329 ± 0.018 ^bcB^
Mix2	7.44 ± 0.51 ^bB^	0.937 ± 0.007 ^bA^	0.67 ± 0.01 ^bcB^	4.96 ± 0.30 ^bB^	4.64 ± 0.25 ^aB^	0.337 ± 0.008 ^bcB^
Mix3	6.59 ± 0.22 ^cB^	0.914 ± 0.004 ^cB^	0.65 ± 0.0 ^cB^	4.31 ± 0.15 ^cB^	3.94 ± 0.15 ^bB^	0.318 ± 0.005 ^cB^
Mix4	4.93 ± 0.75 ^dB^	0.938 ± 0.012 ^bA^	0.68 ± 0.02 ^bB^	3.35 ± 0.43 ^dB^	3.14 ± 0.38 ^cB^	0.342 ± 0.018 ^bB^
**72 h**						
Control	3.03 ± 0.48 ^bA^	0.969 ± 0.016 ^aA^	0.67 ± 0.01 ^aB^	2.03 ± 0.33 ^bA^	1.97 ± 0.29 ^bA^	0.313 ± 0.010 ^aC^
15% Barley	3.35 ± 0.40 ^bA^	0.966 ± 0.022 ^aA^	0.64 ± 0.01 ^bcC^	2.15 ± 0.23 ^bA^	2.07 ± 0.22 ^bA^	0.310 ± 0.007 ^aC^
30% Barley	6.07 ± 1.35 ^aA^	0.945 ± 0.016 ^aA^	0.65 ± 0.01 ^bC^	3.92 ± 0.90 ^aA^	3.70 ± 0.80 ^aA^	0.317 ± 0.011 ^aC^
45% Barley	6.77 ± 0.93 ^aA^	0.946 ± 0.012 ^aA^	0.63 ± 0.02 ^cB^	4.22 ± 0.47 ^aA^	3.99 ± 0.42 ^aA^	0.315 ± 0.024 ^aB^
Control	3.03 ± 0.48 ^cA^	0.969 ± 0.016 ^aA^	0.67 ± 0.01 ^bB^	2.03 ± 0.33 ^dA^	1.97 ± 0.29 ^cA^	0.313 ± 0.010 ^bC^
15% Lentil	5.59 ± 0.35 ^bA^	0.941 ± 0.004 ^bB^	0.65 ± 0.01 ^cC^	3.61 ± 0.25 ^cA^	3.40 ± 0.24 ^bcC^	0.308 ± 0.006 ^bC^
30% Lentil	7.04 ± 1.02 ^aA^	0.932 ± 0.020 ^bB^	0.64 ± 0.01 ^cC^	4.50 ± 0.60 ^bA^	4.21 ± 0.64 ^bA^	0.311 ± 0.010 ^bB^
45% Lentil	8.33 ± 0.68 ^aA^	0.928 ± 0.021 ^bA^	0.72 ± 0.02 ^aA^	5.54 ± 0.51 ^aB^	6.28 ± 2.10 ^aA^	0.385 ± 0.023 ^aA^
Control	3.03 ± 0.48 ^dA^	0.969 ± 0.016 ^aA^	0.67 ± 0.01 ^aB^	2.03 ± 0.33 ^eA^	1.97 ± 0.29 ^eA^	0.313 ± 0.010 ^aC^
Mix1	9.66 ± 0.26 ^bA^	0.922 ± 0.010 ^bA^	0.59 ± 0.02 ^bC^	5.69 ± 0.05 ^cA^	5.25 ± 0.10 ^cA^	0.285 ± 0.017 ^aC^
Mix2	11.90 ± 0.78 ^aA^	0.918 ± 0.019 ^bA^	0.61 ± 0.02 ^bC^	7.19 ± 0.32 ^aA^	6.59 ± 0.17 ^aA^	0.304 ± 0.019 ^aC^
Mix3	10.34 ± 0.75 ^bA^	0.915 ± 0.017 ^bB^	0.62 ± 0.03 ^bC^	6.36 ± 0.36 ^bA^	5.82 ± 0.36 ^bA^	0.308 ± 0.020 ^aB^
Mix4	7.81 ± 0.75 ^cA^	0.912 ± 0.009 ^bB^	0.60 ± 0.02 ^bC^	4.69 ± 0.42 ^dA^	4.27 ± 0.39 ^dA^	0.284 ± 0.021 ^aC^

The statistical analysis was performed separately within each group, and values followed by different lowercase letters in the same column for each group (barley-supplemented, lentil-supplemented, and mixed bazlama samples) are significantly different (*p* < 0.05). ^A–C^ Values with different uppercase letters in the same column indicate significant differences in the corresponding TPA parameters of the each bazlama sample during the 2nd, 24th, and 72nd hours of storage period (*p* < 0.05).

**Table 3:** Color values of the bazlama samples.

	Crumb Color				Crust Color			
Samples	L*	a*	b*	ΔE	L*	a*	b*	ΔE
Control	77.12 ± 0.21 ^a^	0.90 ± 0.01 ^c^	23.22 ± 0.57 ^a^		68.04 ± 0.54 ^a^	7.85 ± 0.35 ^a^	28.96 ± 0.01 ^a^	
15% Barley	73.26 ± 1.12 ^b^	1.37 ± 0.03 ^bc^	22.86 ± 0.23 ^a^	3.91	68.10 ± 1.51 ^a^	7.74 ± 0.42 ^a^	23.27 ± 0.79 ^bc^	5.69
30% Barley	69.40 ± 0.45 ^c^	2.01 ± 0.23 ^b^	23.49 ± 1.07 ^a^	7.80	69.52 ± 0.06 ^a^	7.22 ± 0.58 ^a^	23.46 ± 0.52 ^b^	5.73
45% Barley	65.68 ± 0.39 ^d^	2.79 ± 0.42 ^a^	24.51 ± 0.31 ^a^	11.66	71.44 ± 1.82 ^a^	6.36 ± 0.26 ^a^	22.02 ± 0.03 ^c^	7.87
Control	77.12 ± 0.21 ^a^	0.90 ± 0.01 ^c^	23.22 ± 0.57 ^c^		68.04 ± 0.54 ^a^	7.85 ± 0.35 ^a^	28.96 ± 0.01 ^a^	
15% Lentil	64.77 ± 0.66 ^b^	1.63 ± 0.08 ^bc^	26.04 ± 0.06 ^b^	12.68	66.43 ± 2.39 ^ab^	8.33 ± 1.49 ^a^	24.08 ± 1.24 ^b^	5.16
30% Lentil	61.98 ± 0.96 ^c^	2.35 ± 0.74 ^ab^	28.22 ± 0.93 ^a^	16.01	62.29 ± 2.70 ^bc^	8.99 ± 0.03 ^a^	21.76 ± 1.38 ^b^	9.28
45% Lentil	58.86 ± 1.41 ^d^	3.34 ± 0.18 ^a^	28.99 ± 0.37 ^a^	19.30	59.81 ± 0.80 ^c^	8.90 ± 0.53 ^a^	23.50 ± 2.45 ^b^	9.93
Control	77.12 ± 0.21 ^a^	0.90 ± 0.01 ^c^	23.22 ± 0.57 ^b^		68.04 ± 0.54 ^a^	7.85 ± 0.35 ^b^	28.96 ± 0.01 ^a^	
Mix1	61.54 ± 0.57 ^c^	3.30 ± 0.06 ^b^	25.45 ± 0.64 ^a^	15.92	60.08 ± 2.64 ^c^	9.96 ± 0.01 ^a^	25.23 ± 1.80 ^b^	9.04
Mix2	61.59 ± 0.63 ^c^	3.47 ± 0.09 ^ab^	25.66 ± 0.10 ^a^	15.93	60.54 ± 0.97 ^c^	9.82 ± 0.15 ^a^	28.48 ± 0.17 ^a^	7.77
Mix3	64.52 ± 1.01 ^b^	3.51 ± 0.13 ^a^	25.54 ± 0.52 ^a^	13.07	64.58 ± 0.70 ^b^	7.97 ± 0.52 ^b^	25.03 ± 0.03 ^b^	5.23
Mix4	65.28 ± 0.25 ^b^	3.36 ± 0.00 ^ab^	25.71 ± 0.25 ^a^	12.35	62.82 ± 0.10 ^bc^	9.83 ± 0.06 ^a^	24.32 ± 0.73 ^b^	7.26

The statistical analysis was performed separately within each group, and values followed by different letters in the same column for each group (barley-supplemented, lentil-supplemented, and mixed bazlama samples) are significantly different (*p* < 0.05).

**Table 4:** Protein content, β-glucan content, in vitro GI values of the bazlama samples.

Samples	Protein Content (dwb, %)	β-Glucan (g/100 g Dry Weight)	Glycemic Index (GI)
Control	16.67 ± 0.29 ^a^	0.43 ± 0.04 ^d^	75.16 ± 0.38 ^a^
15% Barley	15.82 ± 0.16 ^b^	0.86 ± 0.01 ^c^	71.30 ± 0.21 ^b^
30% Barley	15.33 ± 0.05 ^b^	1.30 ± 0.01 ^b^	66.68 ± 0.16 ^c^
45% Barley	14.59 ± 0.15 ^c^	1.85 ± 0.02 ^a^	62.27 ± 0.21 ^d^
Control	16.67 ± 0.29 ^d^	0.43 ± 0.04 ^a^	75.16 ± 0.38 ^a^
15% Lentil	18.23 ± 0.06 ^c^	0.37 ± 0.02 ^ab^	70.50 ± 0.13 ^b^
30% Lentil	20.20 ± 0.18 ^b^	0.34 ± 0.03 ^b^	65.27 ± 0.29 ^c^
45% Lentil	22.15 ± 0.09 ^a^	0.14 ± 0.01 ^c^	61.78 ± 0.17 ^d^
Control	16.67 ± 0.29 ^b^	0.43 ± 0.04 ^d^	75.16 ± 0.38 ^a^
Mix1	17.25 ± 0.00 ^a^	1.45 ± 0.00 ^c^	62.74 ± 0.30 ^b^
Mix2	17.00 ± 0.00 ^ab^	1.51 ± 0.01 ^b^	57.24 ± 0.33 ^d^
Mix3	16.31 ± 0.00 ^c^	1.56 ± 0.01 ^b^	58.01 ± 0.36 ^d^
Mix4	15.59 ± 0.00 ^d^	1.62 ± 0.02 ^a^	59.17 ± 0.50 ^c^

The statistical analysis was performed separately within each group, and values followed by different letters in the same column for each group (barley-supplemented, lentil-supplemented, and mixed bazlama samples) are significantly different (*p* < 0.05).

**Table 5:** Phenolic content and antioxidant capacities (DPPH, FRAP, and ABTS methods) of the bazlama samples.

	Samples	Phenolic Content	DPPH	FRAP	ABTS
Free	Control	200.73 ± 0.87 ^d^	33.84 ± 0.67 ^d^	13.32 ± 0.65 ^d^	52.30 ± 0.76 ^d^
	15% Barley	210.41 ± 1.19 ^c^	42.56 ± 0.67 ^c^	21.02 ± 0.66 ^c^	70.23 ± 0.30 ^c^
	30% Barley	220.95 ± 0.88 ^b^	46.42 ± 1.36 ^b^	27.50 ± 0.83 ^b^	85.45 ± 0.93 ^b^
	45% Barley	231.34 ± 2.04 ^a^	54.94 ± 1.05 ^a^	37.51 ± 0.92 ^a^	277.16 ± 2.50 ^a^
Bound	Control	204.33 ± 0.87 ^d^	39.21 ± 0.67 ^d^	36.48 ± 0.90 ^d^	85.84 ± 0.46 ^d^
	15% Barley	232.25 ± 1.74 ^c^	44.58 ± 1.34 ^c^	45.33 ± 0.62 ^c^	166.02 ± 0.60 ^c^
	30% Barley	235.54 ± 1.00 ^b^	56.83 ± 1.71 ^b^	58.55 ± 0.50 ^b^	187.78 ± 0.77 ^b^
	45% Barley	243.95 ± 1.16 ^a^	60.42 ± 1.05 ^a^	65.75 ± 2.02 ^a^	290.34 ± 1.55 ^a^
Total *	Control	405.06 ± 1.71 ^d^	73.05 ± 0.67 ^d^	49.80 ± 0.97 ^d^	138.14 ± 1.22 ^d^
	15% Barley	442.66 ± 2.85 ^c^	87.14 ± 2.01 ^c^	66.35 ± 0.99 ^c^	236.25 ± 0.91 ^c^
	30% Barley	456.49 ± 0.33 ^b^	103.25 ± 0.39 ^b^	86.05 ± 1.33 ^b^	273.23 ± 0.64 ^b^
	45% Barley	475.29 ± 1.87 ^a^	115.36 ± 1.98 ^a^	103.26 ± 2.26 ^a^	567.50 ± 3.76 ^a^
Free	Control	200.73 ± 0.87 ^d^	33.84 ± 0.67 ^d^	13.32 ± 0.65 ^d^	52.30 ± 0.76 ^d^
	15% Lentil	207.37 ± 1.14 ^c^	38.98 ± 1.03 ^c^	28.64 ± 0.78 ^c^	112.43 ± 0.46 ^c^
	30% Lentil	218.77 ± 1.14 ^b^	40.77 ± 1.03 ^b^	34.97 ± 0.76 ^b^	126.94 ± 0.76 ^b^
	45% Lentil	229.40 ± 1.19 ^a^	45.47 ± 1.03 ^a^	49.72 ± 0.45 ^a^	142.13 ± 2.31 ^a^
Bound	Control	204.33 ± 0.87 ^d^	39.21 ± 0.67 ^d^	36.48 ± 0.90 ^b^	85.84 ± 0.46 ^d^
	15% Lentil	212.31 ± 1.19 ^c^	45.70 ± 1.03 ^c^	36.84 ± 0.75 ^b^	120.09 ± 0.91 ^c^
	30% Lentil	221.23 ± 1.83 ^b^	56.22 ± 1.03 ^b^	50.94 ± 9.97 ^a^	144.57 ± 0.30 ^b^
	45% Lentil	233.96 ± 2.30 ^a^	58.68 ± 1.16 ^a^	52.02 ± 0.54 ^a^	295.18 ± 1.21 ^a^
Total *	Control	405.06 ± 1.71 ^d^	73.05 ± 0.67 ^d^	49.80 ± 0.97 ^d^	138.14 ± 1.22 ^d^
	15% Lentil	419.68 ± 2.16 ^c^	84.68 ± 1.03 ^c^	65.48 ± 1.51 ^c^	232.52 ± 0.70 ^c^
	30% Lentil	440.00 ± 0.87 ^b^	96.99 ± 1.94 ^b^	85.92 ± 10.60 ^b^	271.50 ± 0.46 ^b^
	45% Lentil	463.36 ± 2.16 ^a^	104.15 ± 0.78 ^a^	101.74 ± 0.94 ^a^	437.30 ± 2.93 ^a^
Free	Control	200.73 ± 0.87 ^c^	33.84 ± 0.67 ^e^	13.32 ± 0.65 ^e^	52.30 ± 0.76 ^d^
	Mix1	241.23 ± 1.47 ^b^	83.07 ± 1.05 ^b^	53.39 ± 0.77 ^c^	288.90 ± 1.29 ^b^
	Mix2	243.95 ± 1.16 ^a^	88.10 ± 1.37 ^a^	59.86 ± 0.84 ^a^	296.31 ± 1.29 ^a^
	Mix3	241.68 ± 0.88 ^ab^	65.65 ± 1.71 ^d^	56.95 ± 0.91 ^b^	294.11 ± 1.62 ^a^
	Mix4	239.38 ± 2.02 ^b^	68.59 ± 1.04 ^c^	50.33 ± 0.87 ^d^	274.96 ± 1.96 ^c^
Bound	Control	204.33 ± 0.87 ^c^	39.21 ± 0.67 ^e^	36.48 ± 0.90 ^e^	85.84 ± 0.46 ^c^
	Mix1	260.60 ± 1.85 ^a^	88.10 ± 1.37 ^b^	72.14 ± 0.56 ^b^	291.78 ± 0.62 ^b^
	Mix2	263.17 ± 2.82 ^a^	92.22 ± 1.37 ^a^	77.50 ± 0.99 ^a^	299.19 ± 1.24 ^a^
	Mix3	254.78 ± 2.11 ^b^	84.65 ± 1.04 ^c^	58.63 ± 1.79 ^c^	299.81 ± 2.15 ^a^
	Mix4	251.78 ± 1.74 ^b^	80.13 ± 1.04 ^d^	55.79 ± 1.09 ^d^	293.70 ± 1.27 ^b^
Total *	Control	405.06 ± 1.71 ^e^	73.05 ± 0.67 ^d^	49.80 ± 0.97 ^e^	138.14 ± 1.22 ^d^
	Mix1	501.84 ± 2.68 ^b^	171.17 ± 1.98 ^b^	125.52 ± 1.09 ^b^	580.68 ± 0.94 ^b^
	Mix2	507.12 ± 1.68 ^a^	180.31 ± 1.37 ^a^	137.36 ± 1.25 ^a^	595.50 ± 2.34 ^a^
	Mix3	496.46 ± 2.17 ^c^	150.30 ± 0.78 ^c^	115.58 ± 2.33 ^c^	593.92 ± 0.71 ^a^
	Mix4	491.16 ± 1.57 ^d^	148.72 ± 1.04 ^c^	106.12 ± 1.00 ^d^	568.66 ± 1.83 ^c^

The statistical analysis was performed separately within each group, and values followed by different letters in the same column for each group (barley-supplemented, lentil-supplemented, and mixed bazlama samples) are significantly different (*p* < 0.05). Phenolic contents are expressed as mg GAE/100 g dry weight (dw). ABTS: 2,2′-azino-bis (3-ethyl-benzothiazoline6-sulphonic acid); DPPH: 2,2-diphenyl-1-picrylhydrazyl radical scavenging activity; FRAP: Ferric reducing antioxidant power. * The sum of free and bound antioxidant capacities expressed as mg TE/100 g dw.

## Data Availability

The data are contained in the article. Further details will be available upon request.
